# Exploring Individualized Approaches to Managing Vancouver B Periprosthetic Femoral Fractures: Insights from a Comprehensive Case Series Analysis

**DOI:** 10.7759/cureus.53269

**Published:** 2024-01-31

**Authors:** Adrian Cursaru, Mihnea Popa, Bogdan Cretu, Sergiu Iordache, Georgian L Iacobescu, Razvan Spiridonica, Angel Rascu, Bogdan Serban, Catalin Cirstoiu

**Affiliations:** 1 Orthopedics and Traumatology Department, University Emergency Hospital Bucharest, Bucharest, ROU; 2 Orthopedics and Traumatology Department, Carol Davila University of Medicine and Pharmacy, Bucharest, ROU

**Keywords:** risk factors, revision arthroplasty, open reduction and internal fixation, vancouver classification, periprosthetic femoral fractures

## Abstract

The increasing prevalence of periprosthetic femoral fractures, specifically in the vicinity of the hip, has emerged as a significant issue in recent times. Consequently, there is a need for a thorough examination to enhance the effectiveness of management and treatment approaches. The findings of this study emphasize a significant disparity in the occurrence and characteristics of these fractures, and the multiple cases have highlighted the efficacy of various treatment strategies, such as open reduction and internal fixation, as well as the utilization of cortical strut allografts. Furthermore, the study has identified potential risk factors that have an impact on the characteristics of fractures, providing valuable insights that could be crucial in the development of preventive strategies. This study provides a thorough examination of periprosthetic femoral fractures, highlighting the importance of a cohesive treatment algorithm to improve the handling of such fractures. Moreover, it promotes the need for a collaborative endeavor in conducting research in this field, cultivating a more profound comprehension that has the potential to drive progress in therapeutic approaches, ultimately enhancing patient results over an extended period of time. It is crucial that forthcoming research endeavors persist in expanding upon these discoveries, striving towards a unified methodology in tackling this substantial clinical obstacle.

## Introduction

The field of total hip arthroplasty (THA) is undergoing rapid transformation due to several factors, including the increasing age of the population, advancements in surgical methodologies, and the emergence of novel implant materials. Nevertheless, this advancement is not without its own set of obstacles, primarily the rising prevalence of periprosthetic femoral fractures (PFFs) [1,2]. Fractures, whether they occur during surgery or after surgery, impose a considerable clinical burden, frequently resulting in unfavorable outcomes, elevated reoperation rates, and heightened mortality. The objective of this article is to explore the intricacies surrounding PFFs, encompassing their epidemiology and classification systems, as well as the most recent treatment approaches and risk factors. By doing so, this article provides healthcare professionals with a comprehensive resource for effectively managing this complex complication [1,3].

The prevalence of PFFs exhibits considerable variation, with estimates ranging from 1% to 11% for primary THA and up to 18% for revised hip femoral stems. The variability is further compounded by the expanding indications for THA, which currently encompass not only younger patients with elevated functional demands but also older patients with concurrent medical conditions [4,5]. The phenomenon of an aging population has resulted in a notable increase in THA surgeries, which in turn has led to a rise in postoperative PFFs. Research indicates that individuals who are 85 years of age or older are especially susceptible to adverse effects following a hip fracture, including diminished functional outcomes and elevated mortality rates [6,7].

The utilization of classification systems such as the Vancouver classification, along with its more recent incorporation into the Unified Classification System for Periprosthetic Fractures (UCS-PF), has played a crucial role in providing guidance for treatment strategies. Nevertheless, it is important to acknowledge that these systems do have certain limitations. For example, there is a frequent failure to accurately document fractures that are treated without surgery or those that undergo open reduction and internal fixation (ORIF), resulting in an underestimation of the actual occurrence of proximal femoral fractures [8-10].

The selection of implants also significantly influences the incidence of PFFs. Cementless stems exhibit a significantly elevated incidence of intraoperative fractures, approximately 14 times greater than that observed in cemented stems [3,11,12]. Moreover, the design characteristics of cemented stems, such as the choice between taper-slip and composite beam designs, have been demonstrated to have a substantial influence on the likelihood of PFFs, although there is a scarcity of data available on this particular topic [8,10,13].

An additional crucial factor to consider is the influence of sarcopenia and osteoporosis on the elderly population, rendering them more prone to falls and less capable of withstanding impact. Consequently, this elevates the likelihood of experiencing proximal femoral fractures [7,14]. This article aims to examine the present understanding of PFFs, taking into account their growing prevalence, the developing classification systems, the influence of implant selection, and the significance of patient-specific risk factors. This study presents a comprehensive perspective on proximal femoral fractures, equipping healthcare professionals with the necessary resources to effectively address this complex and frequently debilitating condition.

## Materials and methods

This was a retrospective analysis of PFFs that were treated at the clinic of the University Emergency Hospital Bucharest, Bucarest, Romania. The duration of the study spanned from August 2022 to August 2023. The study was approved by the Ethics Council of the Emergency University Hospital Bucharest (approval number: 54231/21.12.2023).

This study comprised a cohort of nine patients diagnosed with Vancouver B2 and B3 fractures. The selection of these cases was deliberate, as they effectively exemplify the intricacies and difficulties inherent in PFFs. The included patients possessed comprehensive clinical and radiographic records, which were examined retrospectively. The study population consisted of individuals of both genders, encompassing a wide range of ages, which is consistent with the main focus of this article. All surgical procedures were conducted by highly experienced senior surgeons at our clinic, possessing extensive expertise in the fields of orthopedics and traumatology.

The specific arthroplasty procedure that each patient had undergone prior to the occurrence of the fracture, as well as the characteristics of the trauma that caused the fracture (low-energy versus high-energy), were considered, as these variables have the potential to impact the outcomes of treatment. The selection between general anesthesia and lumbar anesthesia was determined by considering the patient's personal preference and the complexity of the case. The patients in the study underwent treatment via a lateral approach involving ORIF utilizing a locking compression plate (LCP) that was secured in place using screws and cerclage cables.

The fractures were categorized based on the Vancouver classification system. In the most recent follow-up, the assessment of clinical outcomes was conducted utilizing the Harris Hip Score (HHS), while the evaluation of radiographic outcomes was performed based on specific criteria aimed at determining the degree of fracture healing and the stability of the implant.

The initiation of physical rehabilitation for patients commenced on the day following the surgical procedure, with the initial focus being on assuming sitting positions. Partial weight-bearing was authorized at the end of a two-week period, with a gradual transition to full weight-bearing by the conclusion of six weeks.

Through the implementation of this rigorous methodology, our objective is to make a meaningful contribution to the understanding and management of PFFs. This will ultimately assist clinicians in navigating the intricate and frequently catastrophic nature of this condition, enhancing their decision-making capabilities.

## Results

PPFs occurring at the femoral stem level, specifically classified as Vancouver B fractures, present a multifaceted and demanding clinical situation that frequently requires surgical intervention. Fractures in the vicinity of a prosthetic implant are classified into B1, B2, and B3 subtypes, which are determined by the stability of the implant and the condition of the surrounding bone tissue. The management of these fractures presents a complex challenge due to the presence of a pre-existing implant, which introduces complications in both the diagnostic process and the formulation of an appropriate treatment plan.

The Vancouver B fractures pose a variety of risks that encompass both immediate and long-term consequences. Immediate concerns include pain and immobility, while long-term complications may involve implant failure, non-union, and potentially sepsis. The inclusion of a prosthetic implant introduces an additional level of intricacy, as it can exhibit either stability or looseness, necessitating distinct surgical methodologies. Furthermore, it is important to consider the patient's general health status, age, and presence of other medical conditions, as these factors can have a substantial impact on the results of surgical interventions. Consequently, adopting a uniform treatment strategy that applies to all patients becomes unfeasible.

Due to the significant consequences involved, surgical intervention frequently emerges as the most feasible course of action for the restoration of function and alleviation of pain. The selection of surgical technique for treating Vancouver B fractures is contingent upon the particular type of fracture and may involve internal fixation utilizing plates and screws, cerclages, or revision arthroplasty with an extended prosthesis. Hence, it is imperative to possess a comprehensive comprehension of these fractures, which should be substantiated by radiographic assessment and in accordance with contemporary medical literature, in order to achieve the most effective patient care.

Vancouver B1

PFFs in the Vancouver Bl classification pose distinct challenges that frequently require surgical intervention. Fractures of this nature manifest in the vicinity of a stable stem and can exhibit either displacement or lack thereof. Although conservative management may be an option for certain undisplaced fractures, surgical intervention is typically the preferred approach when the patient is deemed eligible for surgery. There exists a wide array of surgical options, encompassing a spectrum of techniques such as minimally invasive plate osteosynthesis (MIPO) and ORIF. The selection of these methods is contingent upon several factors, such as the imperative for achieving anatomical reduction, the need for cerclaging around the stem, and the potential necessity for structural graft augmentation.

Case 1

**Figure 1:**
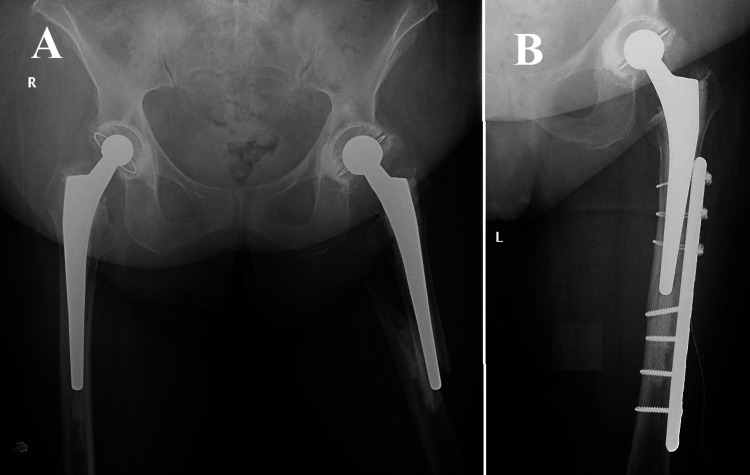
Vancouver B1, Case one One of the instances that exemplify the intricacy of Vancouver B1 periprosthetic femoral fractures involved a 76-year-old individual who had previously received cemented total hip arthroplasty eight years ago. (A) Surgery was required as per the patient's age, cemented stem stability, and fracture characteristics. A specialized plate with distal screws and proximal cerclage wires stabilizes the fracture after an open reduction procedure; (B) Post-surgery radiographs confirm reduction and fixation. These results match the recommended fracture treatment for older patients with stable, cemented prostheses.

The selection of a plate fixation approach (Figure 1) was made due to the stability of the cemented stem in a case concerning a 76-year-old patient with a total hip prosthesis that had been cemented. This decision is consistent with the prevailing literature, which suggests a predilection for plate fixation in approximately 90% of similar instances. The postoperative outcomes of the patient were in line with anticipated results, highlighting the effectiveness of this method for older patients with stable, cemented prostheses. One of the instances that exemplify the intricacy of Vancouver B1 PPF involves a 76-year-old individual who had previously received cemented THA eight years ago. The individual exhibited a positive result following arthroplasty; however, they have recently encountered a periprosthetic Vancouver type B1 fracture as a result of a fall from a standing position. The preoperative radiographic assessment confirmed the B1 categorization of the fracture, as illustrated in Figure 1A. Based on the patient's age, the stability of the cemented stem, and the characteristics of the fracture, it was determined that surgical intervention was required. An open reduction procedure was conducted to realign the fracture, followed by stabilization using a specialized plate that incorporated distal screws and was reinforced with proximal cerclage wires. The radiographs taken after the surgery, as depicted in Figure 1B, verified that the reduction and fixation were successful. These results are consistent with the current recommended approach for treating fractures in older patients who have stable prostheses that are cemented in place.

Case 2

**Figure 2:**
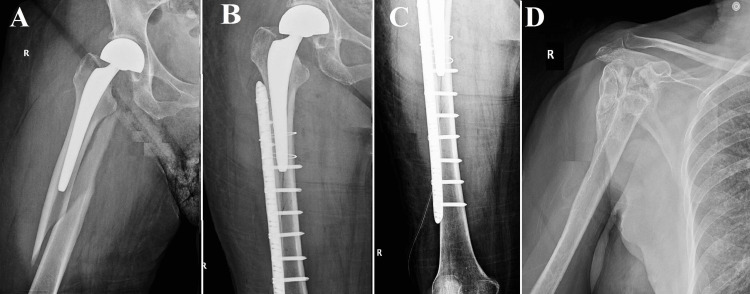
Vancouver B1, Case two (A) A periprosthetic Vancouver type B1 fracture in an octogenarian patient with Alzheimer's disease. Despite initial considerations for non-surgical intervention due to cognitive impairments, surgical intervention become imperative; (B, C) Successful fracture reduction and osteosynthesis following surgery; (D) The humerus's viceous calus

Another case involved an octogenarian patient with a bipolar prosthesis. Due to the patient's advanced age and the specific characteristics of the prosthesis, a meticulous customization of the surgical approach was necessary. The case, presented in Figure 2, highlights the evident complexities associated with managing B1 fractures in patients who have various types of prostheses, including a bipolar prosthesis.

An additional noteworthy instance was that of an octogenarian patient, aged 87, who presented with a multitude of concurrent medical conditions, including Alzheimer's disease, which exerted a substantial impact on the chosen therapeutic strategy. The patient experienced a femoral neck fracture at the age of 85. Due to the patient's advanced age and cognitive impairment, the decision was made to opt for a bipolar prosthesis instead of a total hip replacement. This choice was made with the aim of reducing surgical risks and the likelihood of postoperative dislocation. The patient recently encountered another incident of falling from a standing position, leading to a periprosthetic Vancouver type B1 fracture, as indicated in Figure 2A. In light of the patient's diagnosis of Alzheimer's disease and the concomitant difficulties in providing postoperative care, an initial contemplation was made regarding the implementation of a non-surgical intervention. Nevertheless, given the gravity and specific anatomical site of the femoral fracture, surgical intervention was considered inevitable. The procedures of fracture reduction and osteosynthesis were effectively executed, as evidenced by the visual representations in Figures 2B, 2C. It is noteworthy to mention that approximately 10 years prior, the patient had also experienced a fracture in the proximal humerus region. During that period, the family chose to pursue conservative treatment as a result of the patient's mental condition. Nevertheless, in the case of a femoral fracture, non-operative management was deemed impractical, highlighting the crucial nature of such injuries and the imperative for surgical intervention, even in intricate medical scenarios.

The aforementioned case serves as an illustration of the intricate nature of handling Vancouver B1 fractures in older individuals who have multiple comorbidities, such as cognitive impairments. The statement underscores the importance of adopting a nuanced and personalized treatment strategy that carefully considers the potential risks associated with surgery while also prioritizing the effective management of fractures.

Case 3

**Figure 3:**
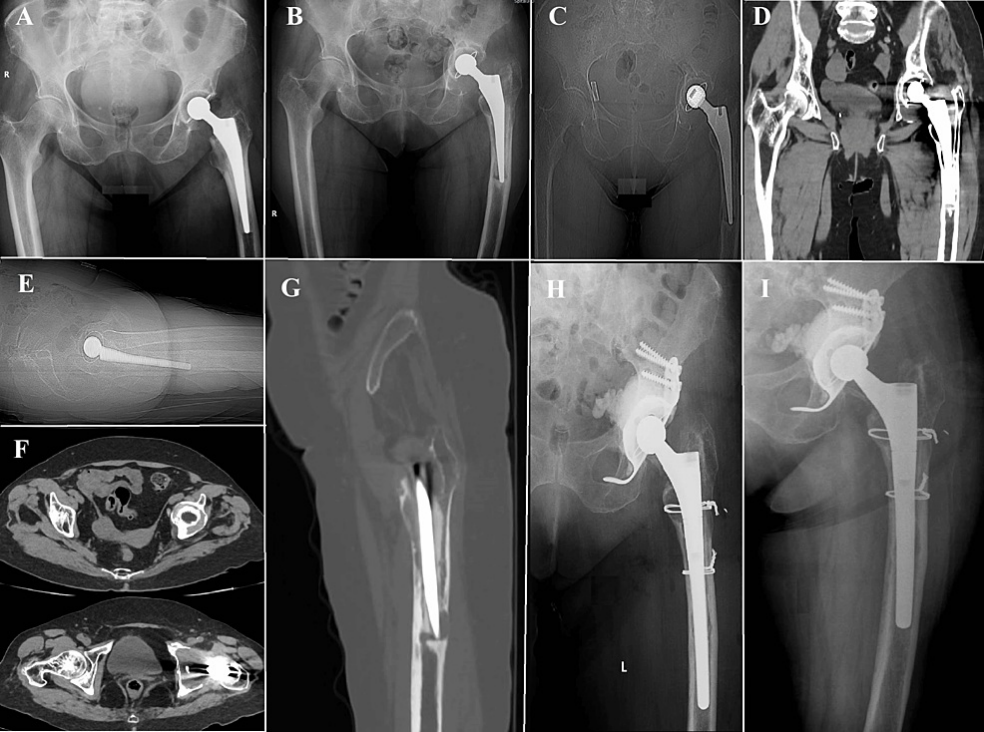
Vancouver B1, Case three (A) Seven years post total hip arthroplasty, slight deterioration; (B) 11 years post arthroplasty, significant deterioration with discomfort; (C-G) CT scans confirm femoral lysis and acetabular loss; (H) Revision hip arthroplasty includes cerclage wires stabilizing the extended stem; (I) One year post-revision, successful healing and prosthesis integration, emphasizing the importance of postoperative monitoring and potential revision procedures for specific fracture characteristics.

A notably arduous circumstance emerged in another patient who underwent a B1 fracture while undergoing the revision of a preexisting prosthesis. The fracture that occurred during the revision procedure introduced an additional level of intricacy, necessitating careful surgical strategizing in order to mitigate the risk of subsequent complications (Figure 3). This particular case serves as an instructive example, underscoring the potential hazards inherent in prosthesis revision surgeries.

This patient, aged 76 years, underwent THA at the age of 65. After a period of seven years following the operation, the radiographic examination revealed slight deterioration without any notable clinical symptoms, as illustrated in Figure 3A. Nevertheless, after a span of 11 years subsequent to the primary arthroplasty, the patient exhibited significant discomfort in the hip region along with notable restrictions in functional abilities. The radiographic assessment conducted at this particular stage (Figure 3B), exhibited significant deterioration observed at both the femoral and acetabular levels. Additional imaging modalities, such as CT scans (Figures 3C-3G), corroborated the presence of notable femoral lysis and considerable loss of acetabular substance. Due to the significant deterioration of the prosthetic device, it was determined that revision hip arthroplasty should be pursued. The patient experienced a Vancouver type B1 periprosthetic femoral fracture during the surgical procedure. In order to tackle this issue, a pair of cerclage wires were employed to provide stability to the extended revision stem, as depicted in Figure 3H. After one year following the revision procedure, the patient exhibited positive results, as indicated by radiographic evidence demonstrating successful healing of the fracture and integration of the prosthesis (Figure 3I). This case serves as a prime illustration of the difficulties and intricacies associated with the management of Vancouver B1 fractures within the framework of revision hip arthroplasty. It highlights the significance of diligent and extended postoperative monitoring for individuals with hip prostheses, particularly in light of the possibility of substantial prosthetic deterioration that may require intricate surgical interventions.

Several fracture characteristics, such as transverse or short oblique patterns, medial comminution, or occurrence over a cemented stem, have the potential to diminish both mechanical and biological healing capabilities. In these particular cases, the suggestion was made to consider a revision procedure involving the elongation of the stem, potentially combined with the use of a structural allograft, as a means to decrease the likelihood of fixation failure.

Vancouver B2

Orthopedic surgery encounters distinctive challenges when dealing with Vancouver B2 fractures. In contrast to B1 fractures, which typically present in the vicinity of a secure femoral stem, B2 fractures are characterized by their occurrence in the presence of an unstable stem, often necessitating a more complex surgical intervention. The intricacy of these fractures is additionally amplified by the requirement for both fracture fixation and prosthesis revision, underscoring the importance of preoperative planning in achieving favorable results.

Case 1

**Figure 4:**
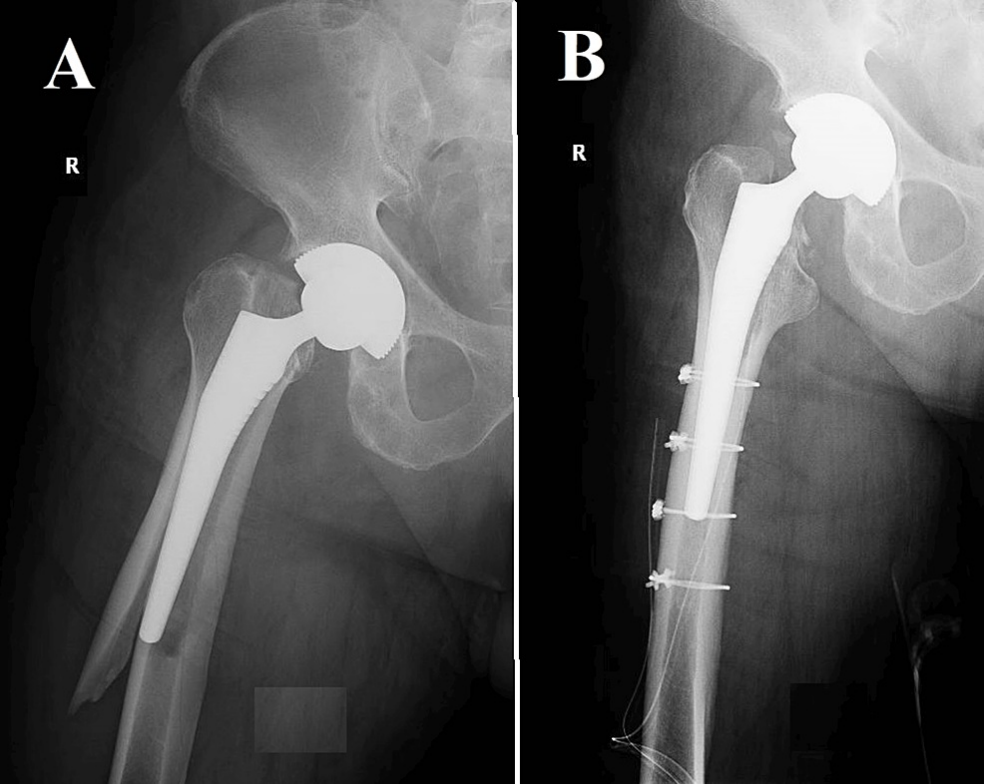
Vancouver B2, Case one (A) The fracture in question is categorized as Vancouver B2 owing to the radiological instability observed in the prosthesis stem. Nevertheless, the bone quality of the patient was remarkably high, enabling a precise anatomical realignment of the fracture during the surgical procedure. Considering the effective decrease in symptoms and the presence of concurrent medical conditions in the patient, it was determined that a more conservative surgical approach would be suitable; (B) The conventional approach for managing these fractures frequently entails the utilization of plate and screw fixation. Nevertheless, in this particular instance, osteosynthesis was accomplished through the utilization of four cerclage wires, deviating from the customary treatment protocol. The patient's postoperative course was without any notable incidents, which serves to underscore the significance of tailoring treatment plans to address intricate Vancouver B2 fractures.

A 56-year-old individual had a medical history of chronic renal pathology since early life, which required prolonged administration of corticosteroids. As a result of the presence of comorbidities, the patient experienced the onset of advanced coxarthrosis at the relatively young age of 48, leading to the necessity of undergoing an uncemented THA procedure. Notwithstanding medical counsel advising otherwise, the individual persisted in participating in athletic endeavors, taking into consideration his comparatively youthful age and vigorous physiological state. The patient, experienced a spiroid fracture during a skiing activity, as illustrated in Figure 4.

The treatment of Vancouver B2 periprosthetic femoral fractures in the Vancouver classification system was frequently complicated by the presence of comorbidities in the patient and the mechanical stability of the current implant.

Case 2

**Figure 5:**
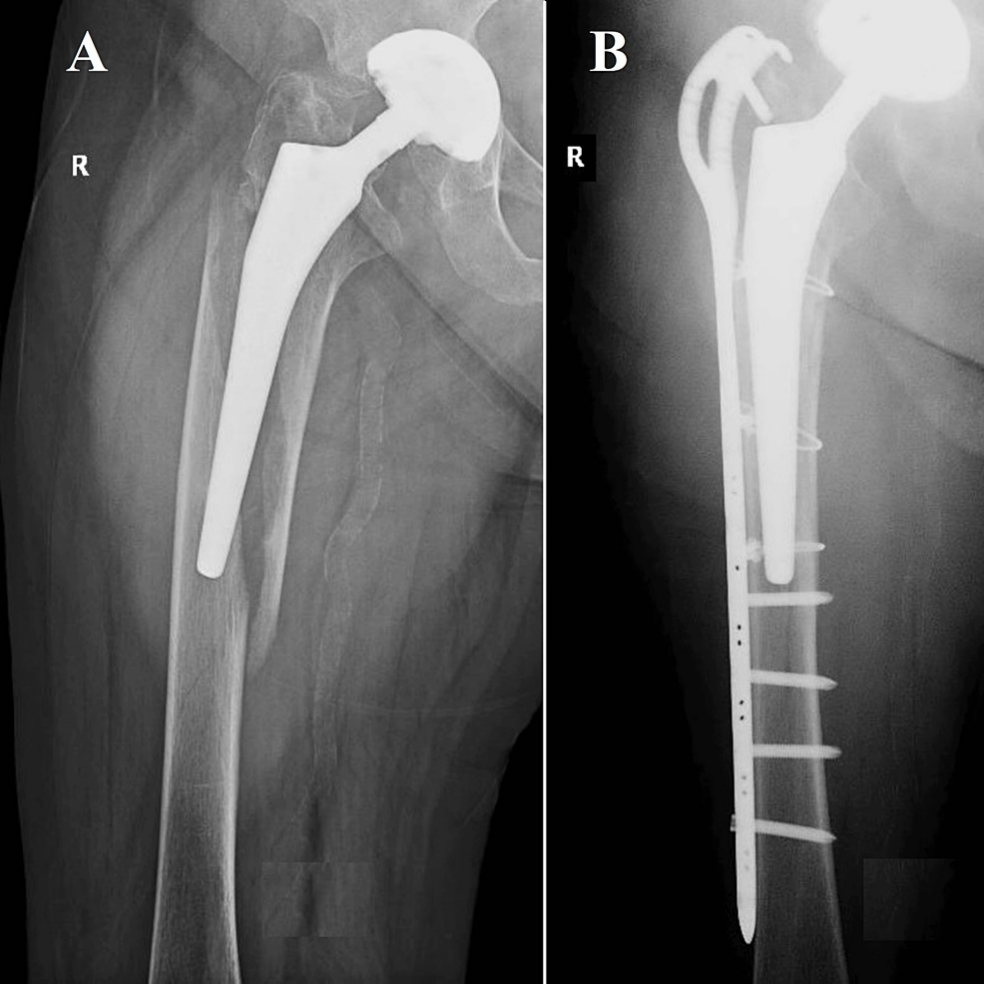
Vancouver B2, Case two (A) Spiroid fracture; (B) Radiological assessment following open reduction and internal fixation (ORIF) using a proximal hook plate, cerclages, and self-locking screws

An octogenarian female patient, approximately 12 years ago, underwent a cemented THA procedure to address coxarthrosis. Subsequently, the patient's medical background became more intricate due to the presence of hypertension, diabetes mellitus, New York Heart Association (NYHA) Class 3 chronic heart failure, and notable cerebrovascular stenosis affecting her carotid arteries. After experiencing an episode of lipothymia, the patient exhibited a multifaceted, lengthy spiral fracture that spanned from the greater trochanter to a location roughly 2 cm below the femoral stem (Figure 5). Typically, the femoral artery was not discernible on conventional radiographic imaging of the hip and femur. The observed visibility indicated the presence of notable vascular pathology. After carefully considering the patient's complex medical history and the unstable nature of the fracture as observed through radiological imaging, it was deemed prudent to exclude the option of a high-risk surgical revision of the femoral component. Alternatively, a surgical approach that was both conservative and efficacious was implemented.

The significance of tailoring treatment plans to the specific needs of patients with Vancouver B2 fractures, especially in geriatric individuals with multiple medical comorbidities, is emphasized by this approach. The postoperative course of the patient has demonstrated positive outcomes, thereby confirming the effectiveness of this individualized approach.

Case 3

**Figure 6:**
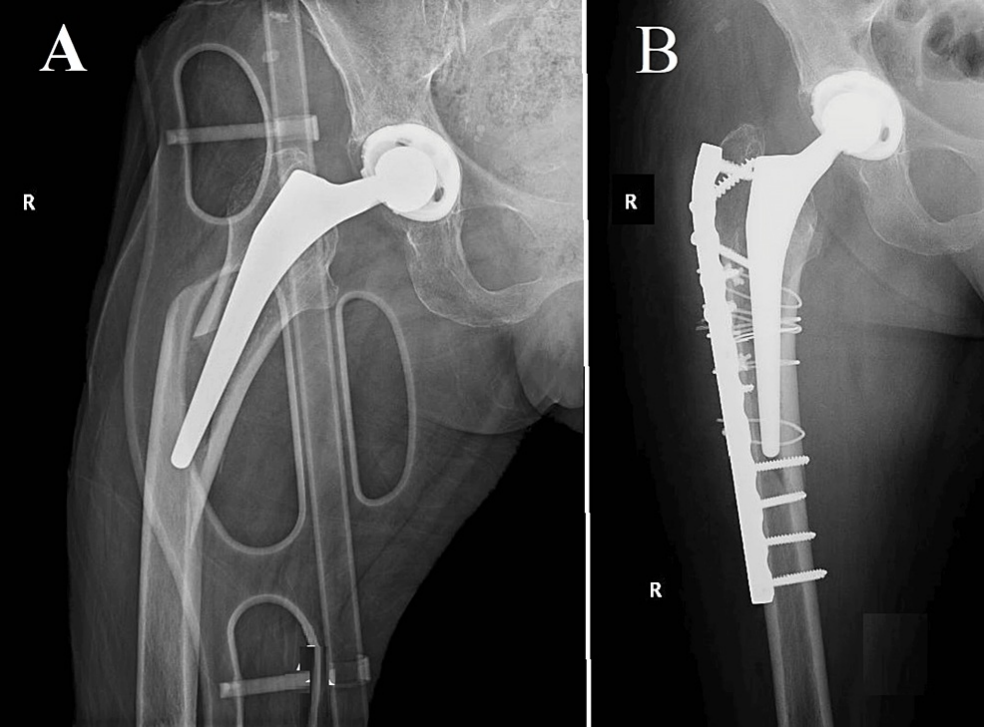
Vancouver B2, Case three (A) A spiroid fracture located at the femur level, accompanied by an unstable femoral stem; (B) The anatomical realignment of the periprosthetic fracture using proximal and distal screws in conjunction with plates to enhance its stability. Additionally, a relatively large number of cerclages have been employed at the plate level to maintain the fracture's position. While this approach does indeed enhance resistance, it also elevates the risk of compromising the vascularization of the bone at this particular site.

A septuagenarian individual, aged 77 years, presented with a seven-year-old unsecured total hip prosthesis. The individual experienced a PFF subsequent to a descent down a set of stairs. The fracture exhibited a spiral pattern and encompassed an intricate "butterfly wing" element, thereby introducing an extra level of intricacy to the already demanding clinical situation (Figure 6). In the specific context of Vancouver B2 fractures, this particular case holds significant importance due to various reasons. The presence of a spiral configuration in the fracture, along with the inclusion of a butterfly wing component, indicated a considerable degree of instability. Consequently, the successful management of such a fracture necessitates meticulous surgical planning and precise execution. Furthermore, the age of the patient and the length of time since the initial hip replacement introduced an additional level of intricacy. Although the uncemented prosthesis presumably attained satisfactory biological fixation over time, the integrity of the adjacent bone stock might have been compromised as a result of osteoporosis associated with aging. Therefore, it was imperative to conduct a meticulous assessment of bone quality during the process of surgical planning in order to guarantee that the selected method of fixation would offer sufficient stability.

Vancouver B3

Vancouver B3 PFFs pose significant challenges within the field of orthopedic surgery. These fractures exhibit an unstable stem and insufficient bone quality, which pose challenges for surgical planning and postoperative recuperation. The limited bone supply frequently hinders the use of simple fixation techniques, leading to the need for more intricate surgical procedures like long stem revision, allograft-prosthetic composites (APCs), or even proximal femoral replacement. Other fixation techniques, such as the use of extramedullary cortical strut grafts, have been recommended to improve stability and facilitate bone healing, especially in situations where there is insufficient bone supply. These grafts are a valuable addition to intramedullary fixation systems, particularly due to the extended duration required for the incorporation and remodeling of such grafts, which can span up to one year. In patients with low demand, the utilization of a distally locked stem could be considered as a potential salvage intervention. Double mobility implants are frequently employed in conjunction with these constructs in order to enhance joint stability. Successfully managing Vancouver B3 fractures necessitates employing a sophisticated strategy that encompasses an in-depth analysis of not only the fracture's specific attributes but also the patient's comprehensive health condition, coexisting medical conditions, and way of life.

Case 1

**Figure 7:**
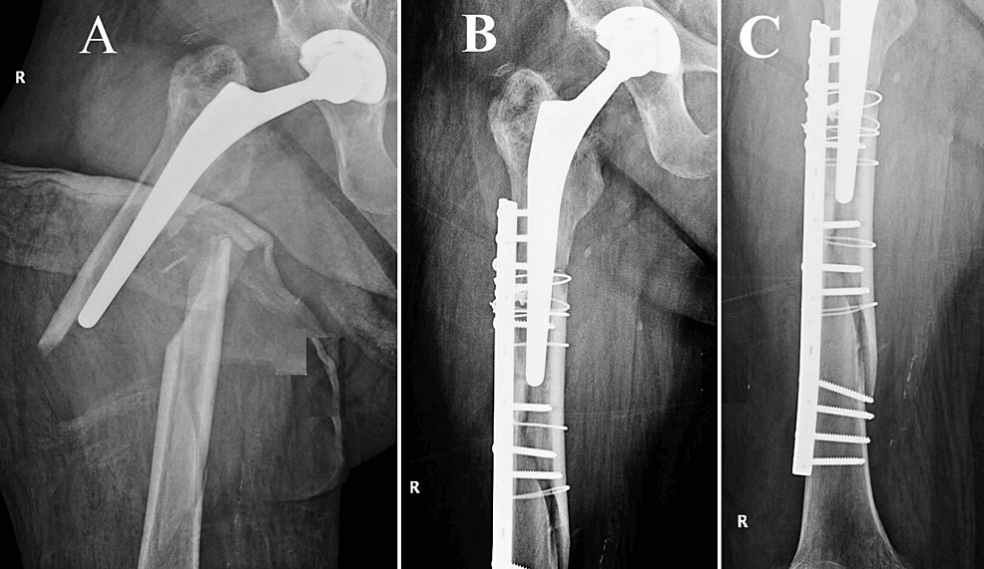
Vancouver B3, Case one (A) A spiroid fracture originating from the trochanteric massif and extending approximately 5 centimeters below the distal end of the femoral stem. The radiological observations pose a particularly complex situation. In this instance of a comminuted fracture, a butterfly fragment become detached, covering a span of approximately two-thirds along the entire length of the fracture. The detachment of the fracture greatly intensifies its instability, presenting a challenging surgical predicament; (B, C) The fracture was anatomically reduced and stabilized using open reduction and internal fixation (ORIF) technique, which involved the application of a self-locking plate supplemented with cables. The selection of this approach was likely made with the intention of optimizing the likelihood of achieving fracture union, while simultaneously minimizing the potential for exacerbating the already delicate nature of the fracture.

A 90-year-old patient had a medical history of an uncemented THA procedure 15 years ago. The presence of morbid obesity in the patient, as evidenced by a body mass index (BMI) of 33, introduced an additional level of intricacy in the process of making surgical decisions. The radiographic results demonstrated a lengthy, fragmented spiral fracture with a butterfly fragment, suggesting substantial instability and a reduction in bone density (Figure 7). Based on the patient's age and obesity, the surgical team made the decision to perform fracture reduction and fixation utilizing a plate and screws, without modifying the femoral component. The objective of this approach was to reduce the potential hazards associated with surgery while ensuring sufficient stabilization.

Case 2

**Figure 8:**
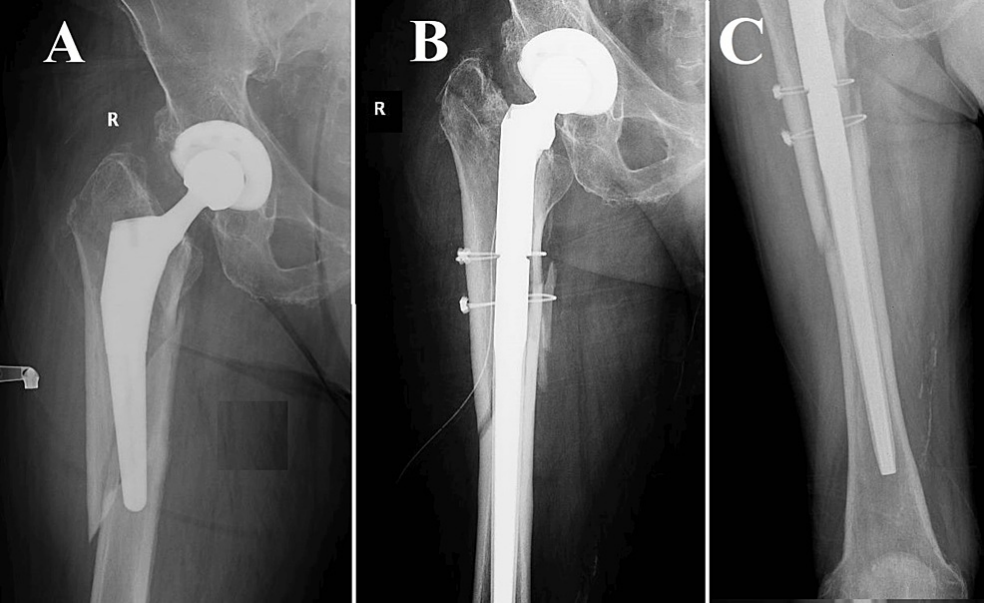
Vancouver B3, Case two Image (A) poses a significant clinical dilemma. The radiographic image demonstrates a comminuted fracture that affects the trochanteric massif and the proximal third of the femur. It is worth noting that there is a considerable degree of telescoping and shortening observed in the femur, which serves as an indication of pronounced instability and displacement. Due to the intricate nature of the fracture and its inherent instability, it is probable that a simple internal fixation would not provide adequate long-term stability and promote fracture union. Within this particular context, the surgical team made the decision to pursue a more assertive course of action, as illustrated in Images (B) and (C). A modification was made to the femoral component by utilizing a longer revision stem in order to improve stability and promote the growth of bone tissue. In order to enhance the stability of the fracture in its anatomical alignment, two cerclages were employed. The utilization of a combined strategy involving the modification of the femoral component and the implementation of cerclages is intended to effectively tackle both the immediate mechanical instability and the enduring biological prerequisites for fracture healing.

The second case pertains to an individual of comparatively younger age, specifically a 74-year-old patient, who maintained an active lifestyle and did not exhibit any notable coexisting medical conditions. The individual in question had previously undergone surgical intervention for coxarthrosis approximately 10 years prior to presentation and had since maintained a physically active lifestyle without experiencing any notable issues (Figure 8). The traumatic incident transpired when the individual was in pursuit of public transportation, leading to a fall from their own level. This particular case serves as a compelling illustration of the complex surgical planning that is necessary for effectively managing Vancouver B3 fractures. The decision to modify the femoral component demonstrates an awareness of the necessity for strong mechanical stability, considering the fragmented and displaced characteristics of the fracture. Cerclages were employed to enhance stability, thereby augmenting the probability of achieving the successful union of the fracture.

Case 3

**Figure 9:**
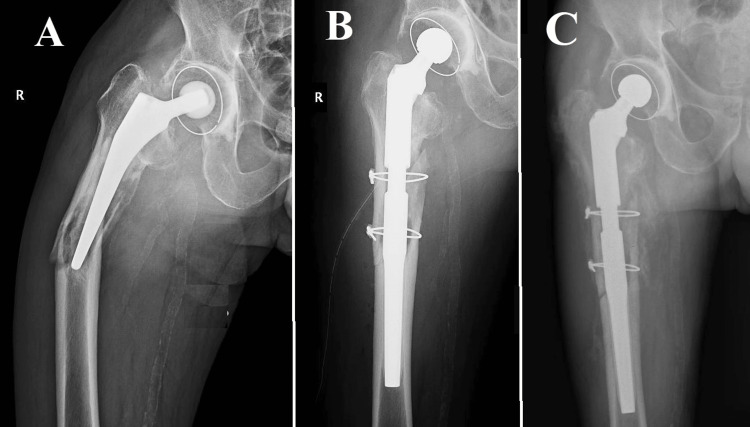
Vancouver B3, Case three (A) A distinctive and demanding clinical situation wherein a spiroid fracture is observed that extends to the apex of the femoral stem. Interestingly, the primary cause of instability in this particular scenario is not solely attributed to the fracture, but rather to the considerable peri-implant lysis that compromised the stability of the pre-existing femoral stem during the occurrence of the trauma. The occurrence of lysis, which refers to the pathological degradation of bone tissue surrounding the implant, further intensified the mechanical instability following the traumatic event. Due to the compromised quality of the tissue and the destabilizing effects caused by the lysis process, it was not feasible to preserve the original stem; (B, C) The surgical team made the decision to perform a revision arthroplasty utilizing a thicker, uncemented modular stem. The utilization of this method facilitated a comprehensive removal of fibrous tissue from the femoral canal until reaching a state of high-quality bone, thereby establishing a more robust base for the subsequent implantation. In order to augment stability and promote the process of fracture healing, two cerclages were administered at the site of the fracture. Every instance mentioned emphasizes the necessity of an individualized strategy for the management of Vancouver B3 fractures. This approach should consider the distinct difficulties and requirements posed by the medical and lifestyle factors of each patient.

The third case involved an octogenarian patient who underwent cemented THA approximately nine years prior to presentation. The patient possessed a notable medical history characterized by the presence of atrial fibrillation and deep vein thrombosis. Radiographic evidence revealed the presence of peri-implant lysis surrounding the femoral component, which was further supported by the patient's reported experience of mild pain during movement (Figure 9). The occurrence of the fracture transpired when the patient experienced a loss of consciousness while rising from a seated position. This case serves as an illustration of the intricate decision-making process involved in the management of complex Vancouver B3 fractures in the context of peri-implant lysis. The surgical approach needed to consider the concurrent issues of acute mechanical instability and compromised bone quality, which led to the requirement for a revision of the femoral component. The implementation of a thicker, uncemented modular stem and the incorporation of supplementary cerclages exemplify a holistic strategy designed to enhance both initial stability and the eventual healing of fractures over an extended period.

## Discussion

The management of PFFs following THA continues to present a multifaceted dilemma within the field of orthopedic surgery. This study, which was carried out at the University Emergency Hospital Bucharest, examines a sample of nine cases in order to gain insights into the complexities of PFFs. The study primarily focuses on the epidemiology, classification, treatment modalities, and patient-specific risk factors associated with these fractures. The absence of algorithms that are universally accepted for the management of PFFs adds further complexity to the clinical environment [10,15].

The current study substantiates the increasing incidence of proximal femoral fractures, particularly among individuals belonging to an aging demographic. The study additionally emphasizes the growing prevalence of "type B fractures," which constitute 90% of fractures occurring after surgery and 70% of fractures occurring during surgery. The Vancouver classification system, which is commonly employed to guide treatment choices, demonstrated a satisfactory degree of agreement among observers for Unified Classification System (UCS) type B fractures (k=0.76). Nonetheless, the differentiation between B1 and B2 fractures presents difficulties as a result of constraints in preoperative X-ray assessments (k=0.64) [16].

The present study highlights the significance of patient-specific factors, such as sarcopenia and osteoporosis, in augmenting the susceptibility of older individuals to proximal femoral fractures. The study found that elderly patients with Vancouver B-type fractures who underwent ORIF had decreased mortality rates and similar hospital stays, regardless of whether the initial stem was press-fit or cemented [17]. This observation is consistent with prior research that has documented an increasing incidence of proximal femoral fractures in the elderly population.

The current study makes a valuable contribution to the ongoing discourse surrounding the effectiveness of ORIF compared to revision arthroplasty in the treatment of Vancouver B2 and B3 fractures. The primary variables assessed encompassed implant subsidence, subsequent revision surgeries, estimated blood loss, and one-year mortality rates. Our study found no statistically significant differences between the groups that received cemented implants and those with press-fit implants, aligning with similar findings in other studies [3,10,17]. Moreover, our study indicates a growing trend in the utilization of cemented and hybrid prostheses, while the adoption of uncemented implants remains consistent [18].

ORIF with anatomic alignment is a viable treatment option for B-type fractures, provided that the cement mantle remains intact and there is an adequate amount of bone stock available. Nevertheless, there is a presence of contradictory evidence regarding the effects of cemented and uncemented stems on outcomes [17,19].

The Vancouver classification system, despite its widespread recognition, possesses certain limitations, notably its incapacity to encompass fractures that are managed without surgical intervention. Additional research is warranted to establish a more comprehensive classification system that has the potential to enhance the effectiveness of treatment. Recent research has indicated that ORIF may yield positive outcomes, particularly in older populations or individuals with comorbidities [20,21]. As an example, a comprehensive analysis revealed that out of a total of 343 B2 fractures, 86.8% of cases were subjected to revision arthroplasty, while 12.6% were treated solely with ORIF. Similarly, among 167 B3 fractures, revision arthroplasty was performed in 95.8% of cases, whereas 4.8% were managed using ORIF [10,22].

The present study provides a comprehensive examination of treatment modalities, specifically focusing on the utilization of cerclage wires and LCPs, which have demonstrated efficacy in particular contexts [10,17]. Customized treatment approaches are of utmost importance in the management of Vancouver B3 fractures, as they necessitate a comprehensive strategy that takes into account both mechanical and biological factors. A noteworthy discovery pertains to the efficacy of cortical strut allografts in the treatment of proximal femoral fractures. The utilization of allografts serves the dual purpose of enhancing mechanical stability and facilitating the process of fracture healing, thereby diminishing the duration necessary for bone union [23,24]. The utilization of these allografts was found to be linked with a relatively low infection rate of 2.0%, underscoring the notion that the complexity of the surgical procedure plays a significant role in determining infection rates [24]. The investigation also documented a notable survival rate of 95.8% for cortical strut allografts during the ultimate follow-up, consistent with prior research that has reported survival rates ranging from 93% to 100% [23,25,26].

The analysis provided in Table 1 is a valuable resource within the dynamic domain of orthopedic surgery. It effectively consolidates various studies that investigate the efficacy of diverse arthroplasty and fixation methods for the treatment of hip fractures [27-29]. Over a period of multiple years and involving a variety of research approaches and cohorts, the table consolidates essential information regarding surgical variables, functional results, and possible adverse events, providing a comprehensive perspective on the present state of investigation in this field. The table elucidates the subtle distinctions among different treatment methodologies, emphasizing factors such as duration of surgery, amount of blood loss, and level of post-operative mobility, which play a crucial role in determining the efficacy of the surgical intervention. Furthermore, it illuminates the rates of survival and complications, offering a well-rounded viewpoint that can inform subsequent research endeavors and clinical judgments [26,30].

**Table 1:** Comprehensive analysis of orthopedic surgery studies providing insights into arthroplasty and fixation methods for hip fractures F: female; M: male; LCP: locking compression plate; LAP: locking attachment plate; ORIF: open reduction and internal fixation; THA: total hip arthroplasty; HA: hip arthroplasty

Pub. Year	Authors	Initial Medical Diagnosis	Type of Arthroplasty	Type of Fixation	Hospital and Surgery Outcomes	Union Rate and Radiological Results	Functional, Mobility, and Disability Outcomes	Complications and Reoperations	Survival Rates
2022	Lv et al. [14]	B1: 15, B2: 66, B3: 106	All THA	Group A: ORIF(B1:15,C:2)/Group B: ORIF+cortical strut allografting (B1:15,C:1)	Mean time to fracture healing: Group A: 5.3 months, Group B: 5.1 months. Average follow-up: 9.8 months	Overall union rate: 96.9%. Group A had one nonunion and one malunion case.	N/A	Group A had one nonunion and one malunion case.	N/A
2022	De Maio et al. [10]	B2+B3:28	Total hip arthroplasty, hemiarthroplasty, or revision arthroplasty.	ORIF using screws and cerclage; bone grafting	Clinical and radiographic results were good; patients returned to ambulation.	Post-ORIF B2 fractures scored higher than B3 fractures.	N/A	satisfactory ORIF results without stem revision	N/A
2015	Solomon et al. [26]	B2: 21	ORIF: 12, Stem Revision: 9	N/A	ORIF mean 390 g, Stem Revision mean: 1502.5 g. ORIF: 21, Revision: 36	Bony union in all patients	ORIF 1 pt: Before fx 5, latest fu 5	ORIF: No infection	96.7% (1-year)
2017	Baum et al, [27]	B2: 57	THA: 53, HHA: 4	N/A	ORIF: 35, Revision and ORIF: 8. ORIF non-union: 1, Revision non-union: 8. Non-union cases: 3 (10.7%). Non-union plate breakage: 2	ORIF non-union: 1, Revision non-union: 8	N/A	ORIF: 6, Revision: 9	N/A
2022	Wall et al., [28]	B1: 12, B2: 2, C: 11, other:3	All THA	LCP-LAP construct	Average age at surgery: 73.3 years; BMI:28.4; Gender: 20 F, 8 M. Average follow-up: 9.8 months	Union achieved in all patients	N/A	Overall complications: 17.9%. Three non-unions, one deep infection, and one revision femoral stem loosening required reoperations.	N/A
2015	Lunebourg et al. [29]	B1: 18, B2: 23, B3: 2	Primary THA or HA	N/A	ORIF: 54, Revision: 7	ORIF B2 straight stem: 4, anatomic stem: 1	ORIF: varied, Revision: varied	ORIF: 0, Revision: 3	N/A
2014	Spina et al. [30]	B1: 30, B2: 7, B3: 11	Not specified	N/A	ORIF: varied, Revision: varied	96.7% (1-year), 88.5% (2-year)	ORIF: 4, Revision: 3	ORIF: 4, Revision: 3	96.7% (1-year), 88.5% (2-year)

This study offers a thorough examination of PFFs, highlighting the intricacies involved in their administration. This emphasizes the necessity of individualized treatment approaches, particularly for Vancouver B3 fractures, and advocates for additional research to enhance classification systems and investigate the possibilities offered by emerging technologies. The findings of this study make a substantial contribution to the current body of knowledge and carry significant implications for future research and clinical practice.

The study conducted possesses certain limitations. The retrospective nature of its design and the small size of its sample may potentially introduce selection bias. Subsequent investigations should strive to conduct comprehensive and forward-looking studies to corroborate the aforementioned findings. Moreover, the influence of nascent technologies such as three-dimensional printing on proximal femoral fracture management has yet to be thoroughly examined, thus necessitating additional research.

## Conclusions

This study examined Vancouver B-type PFFs and their management. It highlighted the rising prevalence of these fractures, particularly in the elderly, and the role of patient-specific factors like sarcopenia and osteoporosis in increasing older adults' risk of proximal femoral fractures. Our study suggests using a comprehensive strategy that takes into account patients' medical and lifestyle characteristics to improve outcomes and reduce mortality.

Despite the insightful findings, the current study is limited by selection bias due to its retrospective nature and small sample size. We recommend more prospective studies to confirm these findings and explore PFF management with emerging technologies. Our study adds significantly to the current body of knowledge, laying the groundwork for future research and potentially changing orthopedic clinical practice.
